# RNA-sequencing analysis reveals betalains metabolism in the leaf of *Amaranthus tricolor* L.

**DOI:** 10.1371/journal.pone.0216001

**Published:** 2019-04-25

**Authors:** Shengcai Liu, Xueli Zheng, Junfei Pan, Liyun Peng, Chunzhen Cheng, Xiao Wang, Chunli Zhao, Zihao Zhang, Yuling Lin, Xu XuHan, Zhongxiong Lai

**Affiliations:** 1 Institute of Horticultural Biotechnology, Fujian Agriculture and Forestry University, Fuzhou, China; 2 Institut de la Recherche Interdisciplinaire de Toulouse, Toulouse, France; Hainan University, CHINA

## Abstract

Amaranth plants contain large amounts of betalains, including betaxanthins and betacyanins. Amaranthin is a betacyanin, and its molecular structure and associated metabolic pathway differ from those of betanin in beet plants. The chlorophyll, carotenoid, betalain, and flavonoid contents in amaranth leaves were analyzed. The abundance of betalain, betacyanin, and betaxanthin was 2–5-fold higher in the red leaf sectors than in the green leaf sectors. Moreover, a transcriptome database was constructed for the red and green sectors of amaranth leaves harvested from 30-day-old seedlings. 22 unigenes were selected to analyze the expression profiles in the two leaf sectors. The RNA-sequencing data indicated that many unigenes are involved in betalain metabolic pathways. The potential relationships between diverse metabolic pathways and betalain metabolism were analyzed. The validation of the expression of 22 selected unigenes in a qRT-PCR assay revealed the genes that were differentially expressed in the two leaf sectors. Betalains were biosynthesized in specific tissues of the red sectors of amaranth leaves. Almost all of the genes related to betalain metabolism were identified in the transcriptome database, and the expression profiles were different between the red sectors and green sectors in the leaf. Amaranth plants consist of diverse metabolic pathways, and the betalain metabolic pathway is linked to a group of other metabolic pathways.

## Introduction

Plant pigments mainly include anthocyanins, betalains, carotenoids, and chlorophylls [1]. Anthocyanins are commonly used as natural colorants [2–5]. However, betalains, which are water-soluble nitrogen pigments, are not only more hydrophilic and have a higher tinctorial strength, they also have physiological functions, including anti-oxidative [6] and anti-cancer [7]. activities. Thus, betalains may be useful for developing novel products relevant to the food and medical industries.

Betalains are mainly existed in Caryophyllales species, with the exception of Caryophyllaceae and Molluginaceae species[8–10], as well as some higher fungi [11, 12]. Betalains and anthocyanins cannot coexist in the same plant [13, 14]. Moreover, betalains have been classified as red (crimson) betacyanins and yellow betaxanthins [15]. There are four main types of betacyanins, namely amaranthin, betanin, gomphrenin, and decarboxybetanin [15–17]. Amaranthin, which is the main betacyanin produced in the genus *Amaranthus*, was first isolated from a plant belonging to this genus [18]. Some published articles have referred to amaranthin as amaranthine [17, 18]; however, in this article, we use amaranthin.

Amaranth (*Amaranthus tricolor* L.) plants are widely distributed in warm and tropical regions worldwide, and are an important source of vital dietary components [19–21]. These plants are cultivated as green vegetables similar to spinach, broccoli, and cabbage. Their mucilaginous leaves are rich in vitamins and minerals [22–24]. They also contain betalain pigments, flavonoids, alkaloids, and other elements [25–28] exhibiting anti-oxidative, anti-cancer, anti-viral, anti-parasitic, and radical-scavenging properties that may be useful for treating certain oxidative stress-related disorders [29–31]. Moreover, amaranths have replaced beets as a source of natural betalains [32, 33]. *Amaranthus* plants are substituted for beet as a source to extract natural betalains[34], because they can be cultivated in more diverse environmental conditions and some amaranth genotypes have a higher biomass and contain more amaranthin than red beets [35, 36].

The classic betalain metabolic pathway includes several enzymes, including polyphenol oxidase (PPO), cytochrome P450 (CYP76AD1/5/6), L- dihydroxyphenylalanine 4,5-dioxygenase (DODA), and glucosyltransferase. However, some of the pathway steps occur spontaneously to ultimately generate betalains [10, 37–43].

Aromatic amino acids, including L-tyrosine, L-phenylalanine, and L- tryptophan, which are primary compounds in the shikimate pathway, are synthesized in plants. In some amaranth species, L-tyrosine is a precursor of the betalain metabolic pathway as well as some phenolic pathways, and is involved in the formation of other aromatic amino acids and auxins[18].

Betalain biosynthesis may require many metabolic pathways, including those related to betalains, flavonoids [44, 45], lignin[18], alkaloid[46]. Additionally, *trans*-cinnamic acid and its hydroxyl and methoxy derivatives can be produced by the tyrosine and phenylalanine biosynthetic pathways. Moreover, *p*-coumaric, ferulic, and 3,4-dihydroxycinnamic acids, which are derivatives of *trans*-cinnamic acid, are the precursors of flavonoids and condensed polyphenols (tannins). Meanwhile, coniferyl and sinapyl alcohols as well as *p*-coumaric acid, are the derivatives of *trans*-cinnamic acid that serve as structural materials for lignin synthesis. Furthermore, lignin synthesis decreases when tyrosine and phenylalanine are increasingly used in the pathway in which L-tyrosine is converted to DOPA and then amaranthin [18]. In amaranth plants, the dihydroindoles of amaranthins might be derived from the dopamine precursors phenylalanine and tyrosine, which are converted to *trans*-cinnamic acid, caffeic acid, and *p*-coumaric acid. A previous examination of the diversity in the color of inflorescences from a *Bougainvillea* species indicated that the accumulation of flavonols is accompanied by a decrease in the accumulation of betalains [45]. Another study determined that L-tyrosine is converted to dopamine following the biosynthesis of an isoquinoline alkaloid [46]. Therefore, L-tyrosine, which competes with other secondary metabolic compounds, is involved in many metabolic pathways that result in betalain biosynthesis.

High-throughput sequencing technology is a viable option for studying molecular mechanisms because it can quickly and efficiently generate a large number of gene sequences and molecular information. For example, this technology has been applied to investigate amaranth plants in terms of flowering *in vitro* [47], as well as nutrition and stress responses [48]. The resulting data may be useful for exploiting and developing new uses for amaranth plants. Meanwhile, cell biological methods may be applied to conduct cell- and tissue-specific analyses of gene expression and physiological activities.

Researchers have analyzed the available genome and transcriptome databases to make considerable advances in the characterization of betalain metabolism. For example, some key genes related to betalain biosynthesis (e.g., *bvMYB1* and *CYP76AD1*) have been identified in the beet genome database[38, 39]. Additionally, researchers have identified the genes encoding CYP76AD5 and CYP76AD6, which catalyze the first tyrosine hydroxylation step, similar to CYP76AD1 [43, 49]. Analyses of transcriptome databases resulted in the identification of the key betalain metabolism genes in pitaya [50], bougainvilleas [51], amaranth[52] and other plants[53, 54]. Xu *et al* [45] described a competitive relationship between betalains and flavonoids in the inflorescences of *Bougainvillea* species. On the basis of these results, researchers have examined and functionally verified key betalain metabolism genes in betalain-containing plant species, including *Opuntia* species [55], *Suaeda salsa*[56], *Portulaca*[57], *Parakeelya mirabilis* [58]. A recent study concluded that catalase-phenol oxidase (CATPO), which exhibits catalase and tyrosinase activities, may be involved in betaxanthin synthesis. Furthermore, the *CATPO* gene was identified, and the encoded protein exhibits PPO activity and affects betaxanthin metabolism in red amaranth (*Amaranthus caudatus*). [59]. From the result, there were other enzymes nor PPO—type tyrosinase involved in the betalains biosynthetic pathway.

However, there has been no systematic study on the relationship between the betalain metabolic pathway and other secondary metabolic pathways. Therefore, we applied high-throughput sequencing technology to sequence the cDNA corresponding to the red and green sectors of amaranth leaves. The subsequent analyses of the GO and Kyoto Encyclopedia of Genes and Genomes (KEGG) databases and quantitative real-time polymerase chain reaction (qRT-PCR) assays revealed some differentially expressed genes (DEGs) related to betalain and flavonoid metabolism. This study revealed a relatively complex network involving the betalain biosynthesis pathway and related metabolic pathways. This network differs from known models. Thus, the data presented herein may be useful for more thoroughly characterizing betalain metabolism.

## Results

### Determination of betalain, flavonoid, chlorophyll, and carotenoid contents in amaranth leaves

**Fig 1:**
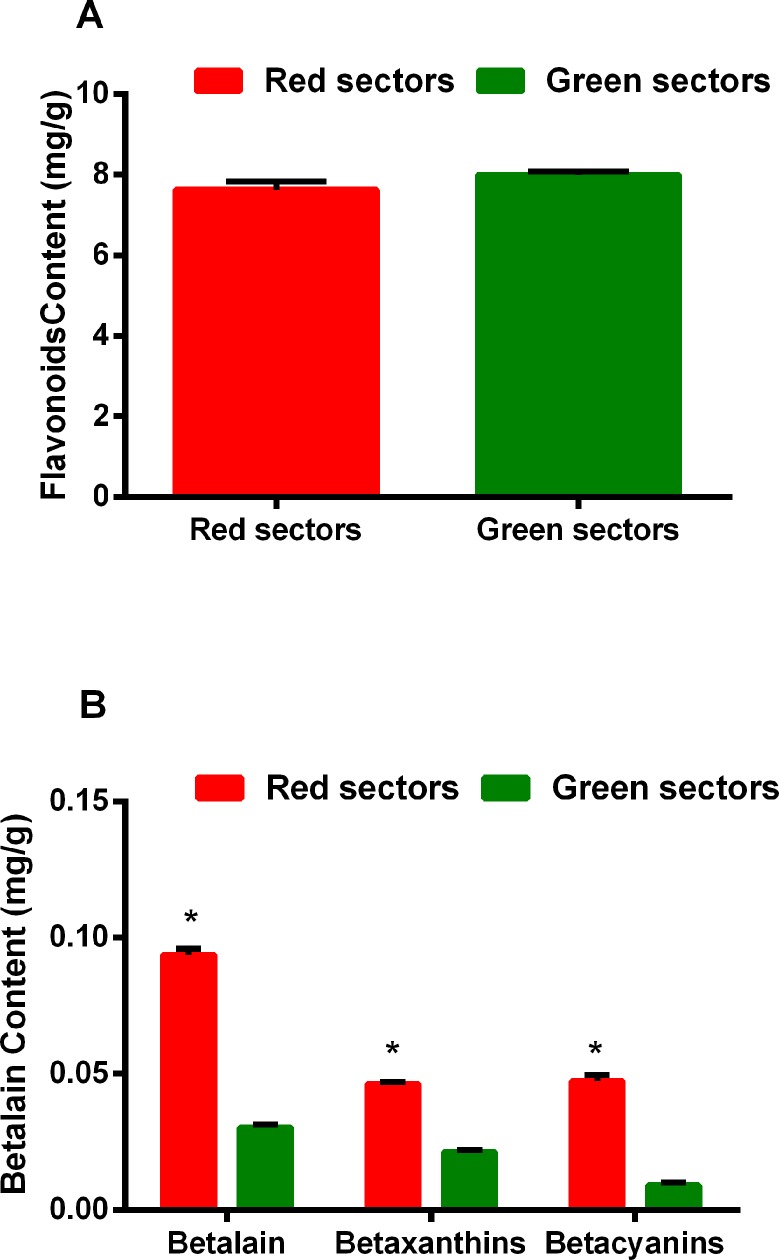
Flavonoid and betalain contents in the green and red sectors of *Amaranthus tricolor* leaves. A: Flavonoid contents; B: Betalain contents.

An analysis of the betacyanin and betaxanthin contents in the green and red leaf sectors indicated these two pigments were more abundant in the red sectors than in the green sectors. In contrast, there were no differences in the flavonoid contents of the two leaf sectors (Fig 1A). Within the red leaf sectors, the betacyanin and betaxanthin contents were similar (betaxanthin to betacyanin ratio of 0.98). However, within the green leaf sectors, betaxanthin was more abundant than betacyanin (betaxanthin to betacyanin ratio of 2.31). Moreover, the total betalain content was 3.09-fold higher in the red leaf sectors than in the green leaf sectors. Furthermore, the betacyanin and betaxanthin contents were 5.15-fold and 2.20-fold higher in the red leaf sectors than in the green leaf sectors, respectively (Fig 1B).

**Fig 2:**
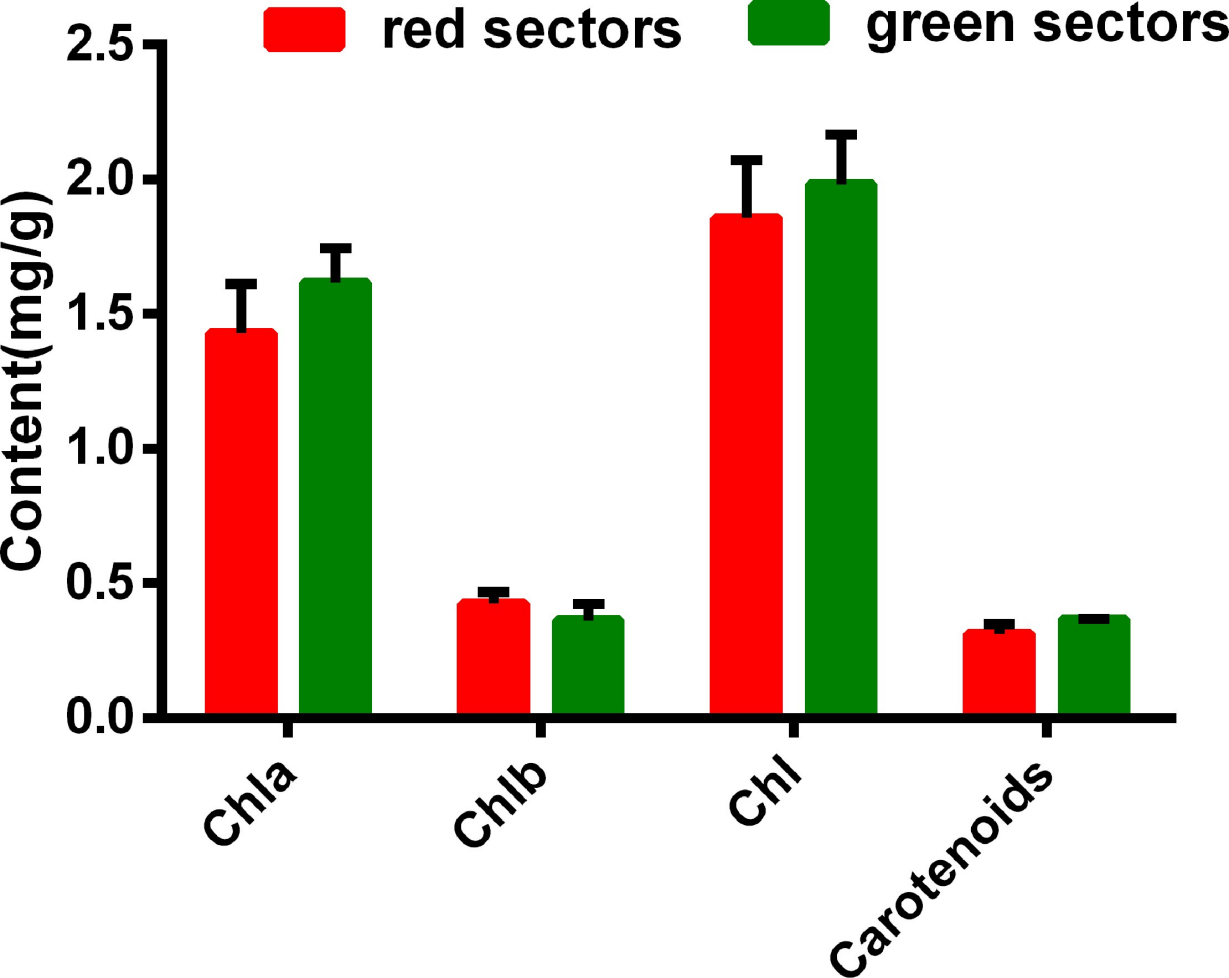
Chlorophyll and carotenoid contents in the red and green sectors of *Amaranthus tricolor* leaves.

However, an analysis of the chlorophyll and carotenoid contents in the red and green leaf sectors revealed a lack of differences between the two sectors (Fig 2). Perhaps betalains are hydrophilic pigments, they accumulate in leaves only in the epidermal subcells on the abaxial surface. Chlorophyll and carotenoids are normally synthesized in the other leaf tissues and cells. Therefore, photosynthates accumulate during plant growth and development, and the associated metabolic activities are influenced by photosynthetic capacity.

### Transcriptome sequencing data assembly of *A*. *tricolor*

Because betalains were present only in the red sectors of amaranth leaves, we extracted RNA separately from the red and green leaf sectors to generate cDNA libraries for sequencing. After removing adapter sequences, ambiguous reads, and low-quality reads, approximately 15.08 Gb high-quality clean reads were obtained, with 29,914,176 reads (7.53 Gb) and 29,913,707 reads (7.53 Gb) corresponding to the red and green leaf sectors, respectively. The raw data were deposited in the NCBI Sequence Read Archive database https://www.ncbi.nlm.nih.gov/sra/SRR5930345 (accession number: SRR5930345). The generated sequencing data are summarized in Table 1. The Q30 percentages (sequencing error rate < 0.1%) for the red and green leaf sectors were 93.58% and 93.37%, respectively. Additionally, the GC contents for the red and green leaf sectors were 43.19% and 42.32%, respectively. These values indicated that the quantity and quality of the sequencing data were sufficient to ensure an accurate sequence assembly with adequate transcriptome coverage.

**Table 1:** Statistic data of transcriptome *A*. *tricolor*.

Samples	Clean reads	Clean bases(bp)	Q30(%)	GC(%)
Red sector	29914176	7538372352	93.58	43.19
Green sector	29913707	7538254164	93.37	42.32

All high-quality reads were assembled *de novo* using the Trinity program, which resulted in 9,414,697 contigs, with a total length of 407,232,755 bp, a mean length of 43.26 bp, and an N50 of 44 bp. On the basis of the paired-end sequence data, 151,663 transcripts were generated, with a total length of 180,734,750 bp, a mean length of 1191.69 bp, and an N50 of 2,168 bp. Transcripts were assembled into 80,026 unigenes, with a total length of 53,799,761 bp, a mean length of 672.28 bp, and an N50 of 1,202 bp. The length distribution of the contigs, transcripts and unigenes was demonstrated in Table 2 and S1 Fig.

**Table 2:** Statistical data of transcriptome sequencing data assembly.

Length Range	Contig	Transcript	Unigene
201–300	9,359,546(99.41%)*	38,288(25.25%)	33,504(41.87%)
301–500	23,738(0.25%)	26,429(17.43%)	19,513(24.38%)
501–1000	15,914(0.17%)	25,385(16.74%)	12,773(15.96%)
1001–2000	9,789(0.10%)	30,431(20.06%)	8,456(10.57%)
>2000	5,710(0.06%)	31,130(20.53%)	5,780(7.22%)
Total Number	9,414,697	151,663	80,026
Total Length	407,232,755	180,734,750	53,799,761
N50 Length	44	2,168	1,202
Mean Length	43.26	1191.69	672.28

The number and percentage with ‘*’ in the table indicate the contig length range from 0 to 300 bp

### Unigene annotation and analysis of amaranth leaf

To annotate genes and predict the encoded proteins, all assembled unigene sequences were used as queries in a BLASTX search (*E*-value < 1 × 10^−5^) of the plant proteins in the Nr, Swiss-Prot, KEGG, COG, and KOG databases. A total of 34,350 significant BLAST hits (42.92% of all unigenes) were obtained (Table 3), with 33,893 unigenes (42.35% of all unigenes) annotated according to the Nr database and 22,508 unigenes (28.13% of all unigenes) annotated according to the Swiss-Prot database. Unigenes with lengths of 300–1,000 bp and > 1,000 bp accounted for 12,017 (34.98%) and 12,664 (36.87%) of the 34,350 annotated unigenes, respectively. Unigenes with lengths of 300–1,000 bp and > 1,000 bp accounted for 11,894 (35.09%) and 12,647 (37.31%) of the unigenes annotated according to the Nr database, respectively. Unigenes with lengths of 300–1,000 bp and > 1,000 bp accounted for 7,712 (34.26%) and 8,962 (39.82%) of the unigenes annotated according to the Swiss-Prot database, respectively. Many genes with unknown functions were detected in the transcriptome database.

**Table 3:** Statistical data of unigene annotation.

Anno_Database	Annotated_Number	300< = length<1000	length> = 1000
COG_Annotation	10,365	2,915	4,961
GO_Annotation	20,677	7,111	7,488
KEGG_Annotation	13,961	4,751	5,054
KOG_Annotation	18,882	6,040	7,707
Pfam_Annotation	21,866	6,730	10,742
Swissprot_Annotation	22,508	7,712	8,962
nr_Annotation	33,893	11,894	12,647
All_Annotated	34,350	12,017	12,664

### Identification of genes participating in the betalains metabolism from the transcriptome database

On the basis of previous investigations by Teng et al. [59] and Zheng et al[52], we identified the unigenes involved in betalain metabolism in the transcriptome following a comparison of these gene sequences by using BLASTX (E-value < 1 × 10^−5^). We identified DEGs between the red and green leaf sectors based on the unique reads with FC ≥ 2 and FDR < 0.01. Of these DEGs, *CATPO* (c36298.graph_c0 and c37003.graph_c0), *CYP76AD1* (c35434.graph_c0), *DODA* (c10239.graph_c0 and c20970.graph_c0), cyclo-DOPA 5-O glucosyltransferase (*DOPA5-GT*) (c18956.graph_c0), betanidin 5-*O*-glucosyltransferase (*B5-GT*) (c10712.graph_c0), betanidin 6-*O*-glucosyltransferase (*B6-GT*) (c28414.graph_c0), and *MYB* (c12243.graph_c0, c24997.graph_c1, and c45546.graph_c0) were more highly expressed in the red leaf sectors than in the green leaf sectors. In contrast, the *CATPO* (c37603.graph_c0), *PPO* (c13148.graph_c0), and *tryptophan decarboxylase* (*TyDC*) (c29034.graph_c0) expression levels exhibited the opposite trend. Moreover, we did not detect *CYP76AD5/6* in the transcriptome database.

### Functions annotation of differentially expressed genes

**Fig 3:**
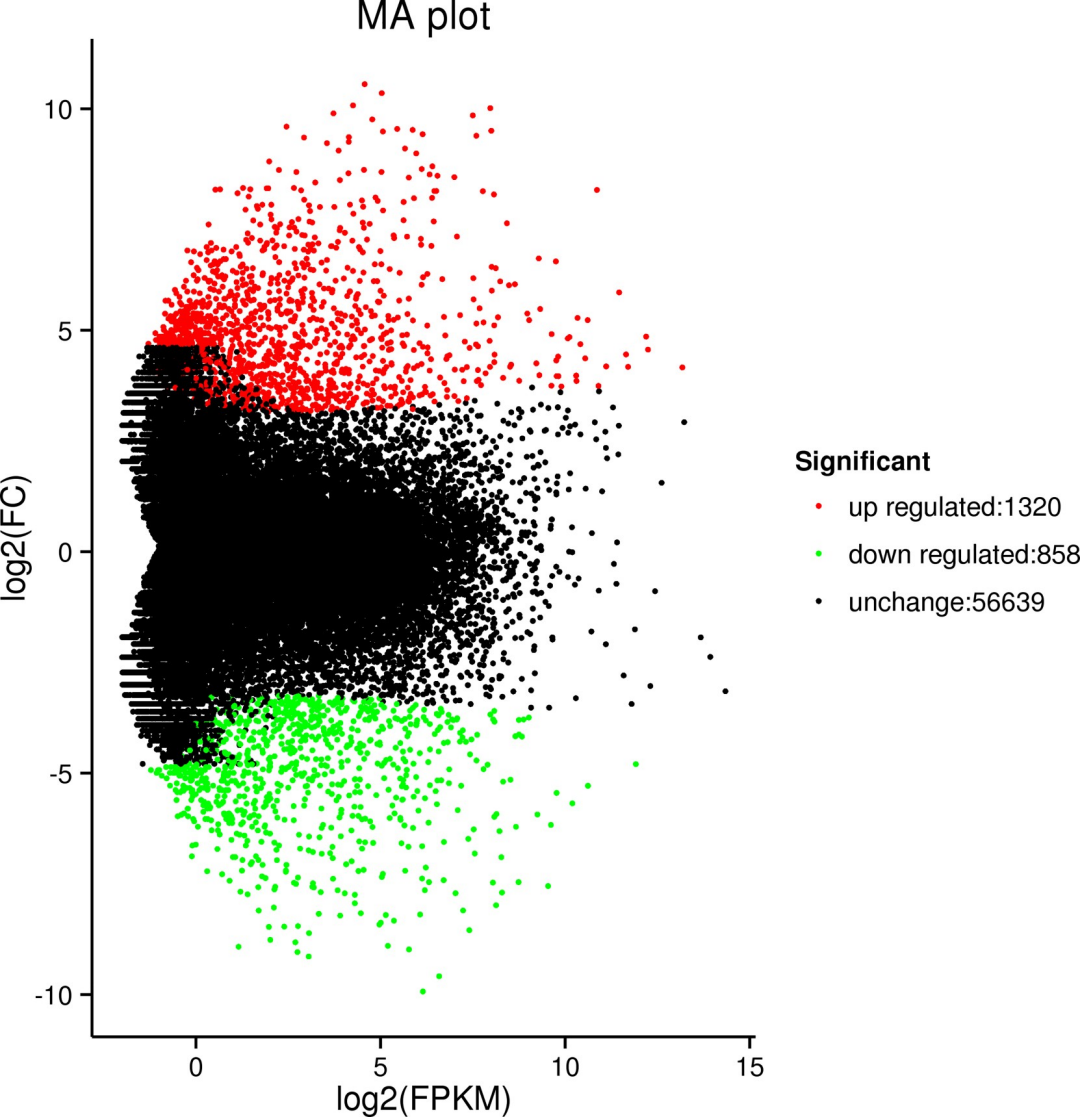
MA map of differentially expressed genes. Each dot in the MA diagram of differentially expressed genes represents a gene. Abscissa is A value. log2(FPKM) represents the logarithm of the difference multiple of gene expression between the two samples. The ordinate is M value. log2(FC), which is the logarithm of the multiple of gene expression difference between the two samples, used to measure the difference of expression. The different colour dots represent the genes expression differences. Amongst, the green, the red and the black dots represent genes with down-regulated expression, up-regulated expression, and no significant expression differences, respectively.

**Fig 4:**
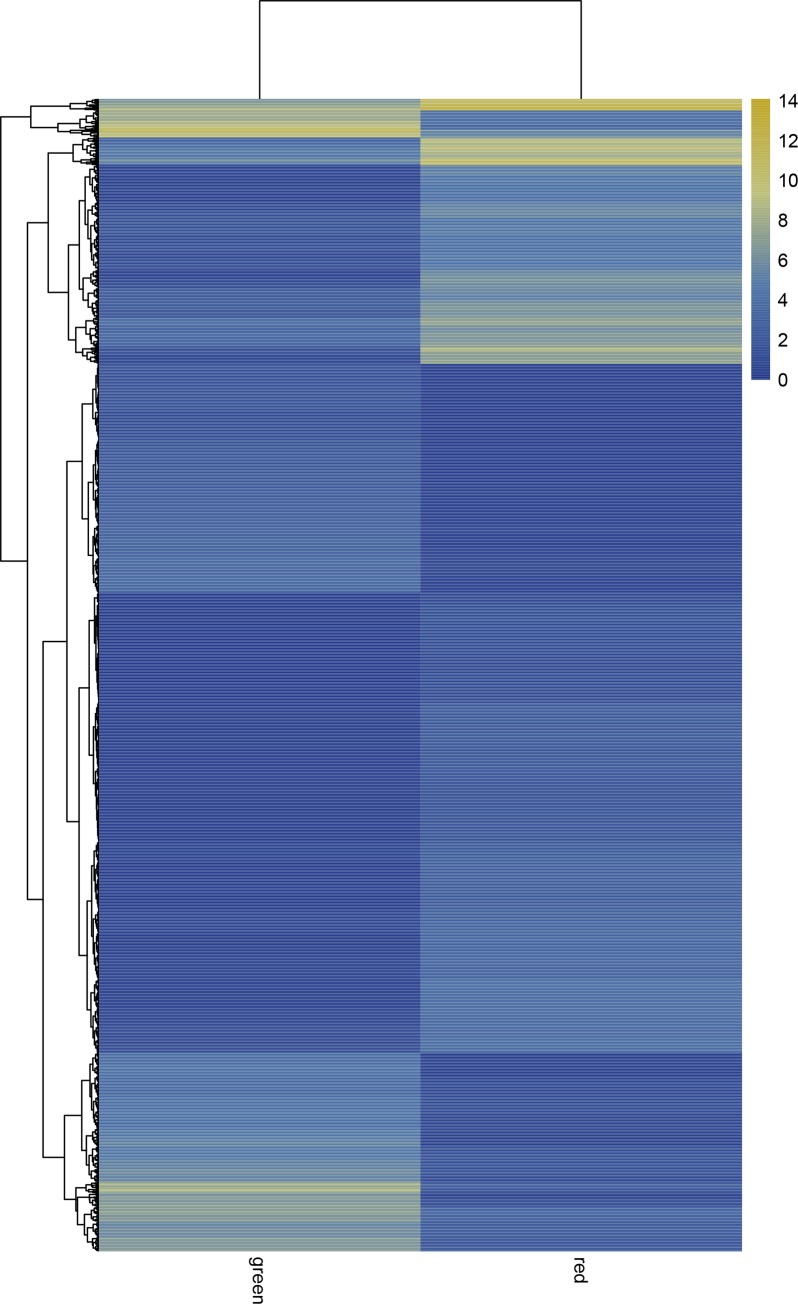
Cluster diagram of expression patterns of differentially expressed genes. The columns represent red and green sectors, and different rows represent different genes. Color represents log 2(FPKM).

We identified 2,178 unigenes that were differentially expressed between the green and red leaf sectors, including 1,320 and 858 genes whose expression levels were respectively up- and down-regulated in the red leaf sectors (Fig 3). Hierarchical clustering analysis was performed on the screened differentially expressed genes. The genes with the same or similar expression profiles were clustered (Fig 4).

All DEG sequences were used as queries in a BLASTX search (E-value < 1 × 10^−5^) of the plant proteins in the Nr, Swiss-Prot, KEGG, COG, and KOG databases. A total of 1,580 (72.54%) unigenes were annotated.

### GO annotation

**Fig 5:**
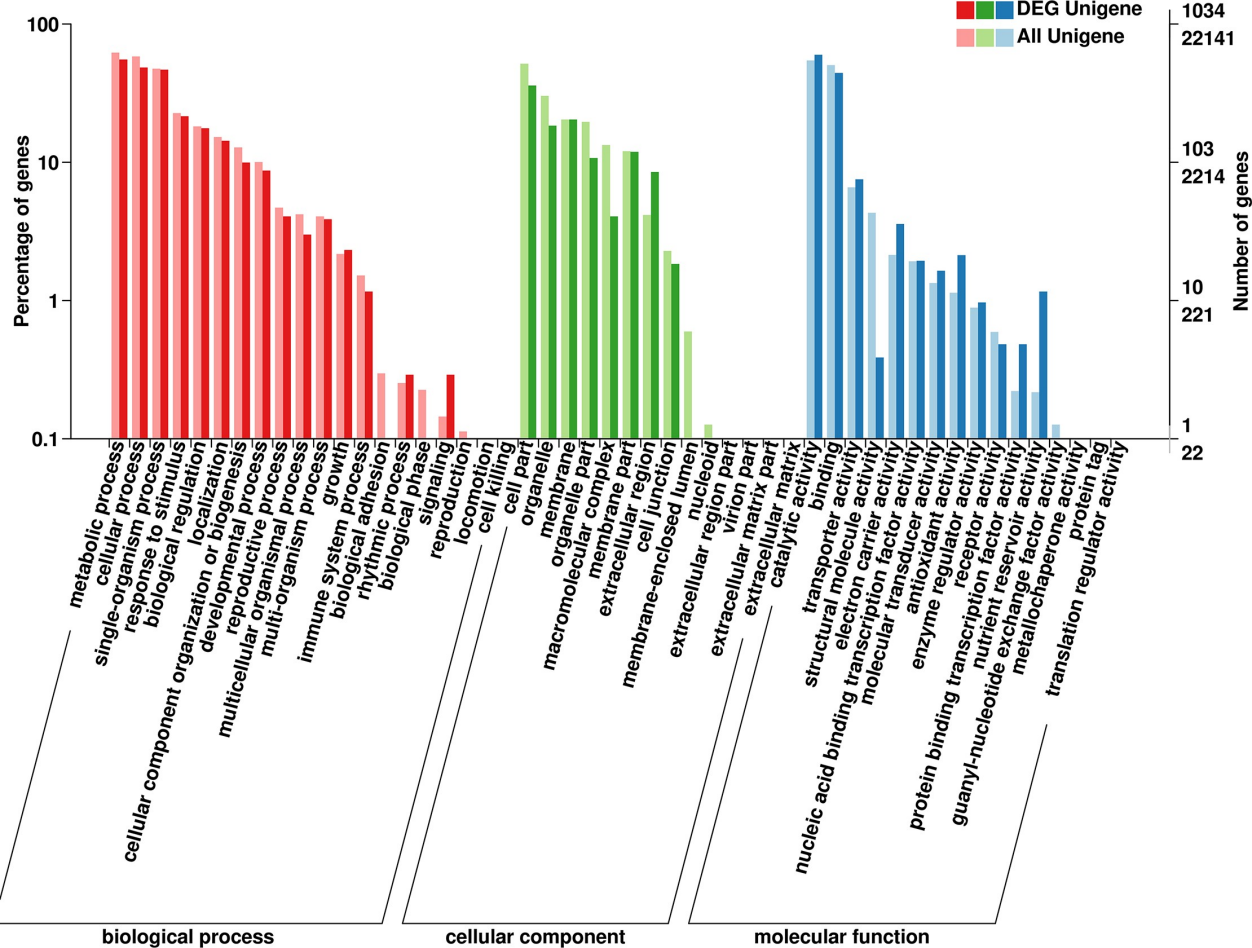
GO categories.

921 DEGs were assigned to the three main GO categories and 50 sub-categories (functional groups). A large proportion of the genes in the biological process category belonged to the “metabolic process” (516), “cellular process” (438), and “single-organism process” (427) sub-categories. Genes belonging to the “cell part” (321), “membrane” (181), and “organelles” (159) sub-categories were heavily represented among the genes in the cellular component category. Meanwhile, “catalytic activity” (564) and “binding” (398) were the dominant sub-categories in the molecular function category. Within each sub-category, the number of genes functionally annotated at each developmental stage was similar to the total number of annotated genes (Fig 5).

Meanwhile, we executed the topGO to enrich GO terms related to betalain metabolism in each of the three main categories (biological process, cellular component, and molecular function). After Kolmogorov-Smirnov test (KS), the 10 most significantly were enriched (S1 Table). The order of the biological process terms was as follows: “mitotic cell cycle”, “sister chromatid cohesion”, “DNA recombination”, “embryo sac egg cell differentiation”, “cellular protein modification process”, “photosynthesis”, “light harvesting”, “intracellular protein transport”, “regulation of cell cycle”, “RNA processing”, and “double-strand break repair”. The order of the cellular component terms was as follows: “plastoglobule”, “integral component of membrane”, “chloroplast thylakoid membrane”, “photosystem II oxygen evolving complex”, “extracellular region”, “photosystem I”, “chloroplast thylakoid”, “cytosolic large ribosomal subunit”, “chromosomal part”, and “PSII associated light-harvesting complex II”. The order of the molecular function terms was as follows: “chlorophyll binding”, “oxidosqualene cyclase activity”, “2-alkenal reductase NAD(P) activity”, “structural constituent of ribosome”, “RNA methyltransferase activity”, “magnesium-protoporphyrin IX monomethyl ester (oxidative) cyclase activity”, “metal ion transmembrane transporter activity”, “non-membrane spanning protein tyrosine kinase activity”, “3′–5′ exonuclease activity”, and “oxidoreductase activity”.

### COG annotation

The 758 DEGs with matches in the COG database were classified into 22 COG categories (S2 Fig). “General function prediction only” represented the largest group (135; 17.81%), followed by “replication, recombination, and repair” (79; 10.42%), “transcription” (76; 10.03%), “signal transduction mechanisms” (70; 9.23%), and “secondary metabolite biosynthesis, transport, and catabolism” (67; 8.84%).

### KEGG annotation

**Fig 6:**
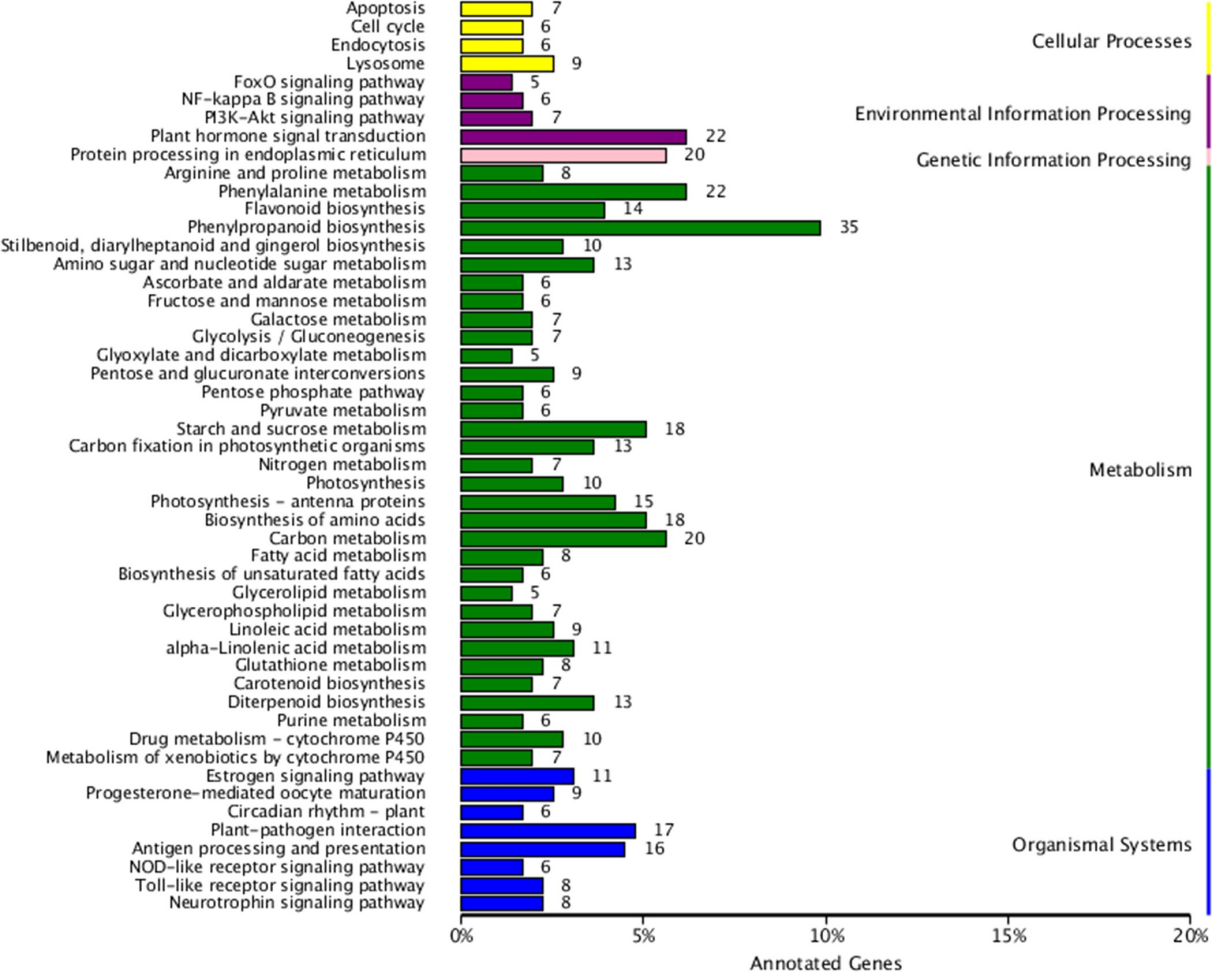
KEGG pathways associated with differentially expressed unigenes.

A total of 521 DEGs were assigned to the following five KEGG biological pathways: “containing cellular processes”, “environmental information processing”, “genetic information processing”, “metabolism”, and “organismal systems” (Fig 6). These KEGG pathways included those related to “phenylpropane synthesis” (35 unigenes), “plant hormone signal transduction” (22 unigenes), “phenylalanine metabolism” (22 unigenes), “flavonoid biosynthesis” (14 unigenes), and “photosynthesis (including antenna proteins)” (25 unigenes). These results indicated that amaranth plants comprise many metabolic pathways, which may influence diverse metabolic activities, including those related to betalains.

### Analysis of DEGs with the MapMan program

According to *A*. *thaliana* metabolism pathways, 35 identified DEGs were related to the metabolism of various hormones, including abscisic acid (ABA), cytokinins, jasmonic acid, salicylic acid (SA), ethylene, and gibberellic acid (GA) (S2 Table). A classification of these unigenes with the MapMan program revealed six unigenes were related to ABA, six unigenes were related to cytokinins, nine unigenes were related to GA, five unigenes were related to jasmonic acid, and seven unigenes were related to SA. Thus, these findings suggest that these hormones may contribute to betalain metabolism, which is consistent with the results of one of our earlier investigations[52]. These hormones might be involved in the betalains metabolism.

Thirty different expression unigenes were involved in photosynthesis, participating in photosystem I (c31077.graph_c0, c34447.graph_c0, c34173.graph_c0, and c34156.graph_c0), photosystem II (c34182.graph_c0, c34213.graph_c0, c34127.graph_c0, c20726.graph_c0, c32263.graph_c0, c34124.graph_c0, c26493.graph_c0, c34159.graph_c0, c38043.graph_c0, c36001.graph_c0, c34319.graph_c0, c34651.graph_c0, c34168.graph_c0), electron transport (c34751.graph_c0, c10825.graph_c0, c12313.graph_c0, c34759.graph_c0, c40314.graph_c0, and c34725.graph_c0), and cingvin cycle (c34412.graph_c0, c34489.graph_c0, c34136.graph_c0, c34161.graph_c0, c37315.graph_c0, c39980.graph_c0, and c34820.graph_c0). (Showed in S3 Table)

In the meanwhile, 8 differential expression unigens were related to sucrose and starch metabolism, including starch synthesis (c11310.graph_c0 and c38113.graph_c0), starch degradation (c36568.graph_c0 and c30136.graph_c0), and sucrose degradation (c26596.graph_c0, c10942.graph_c0, c33445.graph_c0, and c36362.graph_c0) (S4 Table). The genes related to sucrose and starch metabolism expressed differently. Very less starch was found in the leaf cell of red sectors, which pointed to either weak photosynthetic capacity in the red regions of the leaf, or starch degradation into soluble sugar as glucoside that was a key functional group of betalains.

#### Expression profiles of unigenes analysed by qRT-PCR

**Fig 7:**
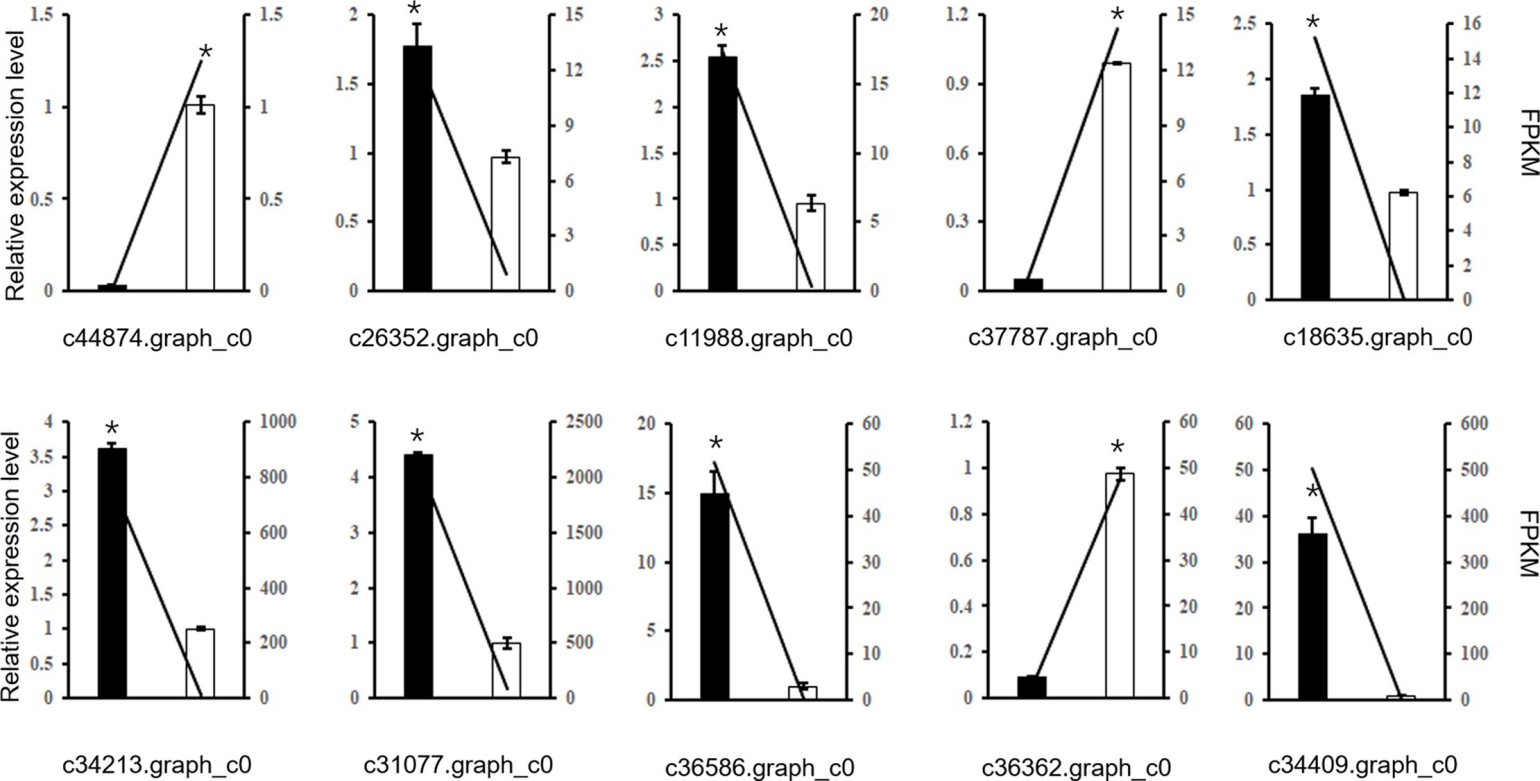
Verification of the accuracy of the RNA-sequencing data in *Amaranthus tricolor* leaves.

We completed a qRT-PCR assay to analyze the expression levels of 10 significant DEGs related to photosynthesis, phenylpropane synthesis, plant hormone signal transduction, and phenylalanine metabolism to validate the accuracy of the RNA-Seq data. The expression patterns of these genes according to the qRT-PCR were consistent with the RNA-Seq data, implying that the RNA-Seq data were suitable for subsequent analyses (Fig 7).

**Fig 8:**
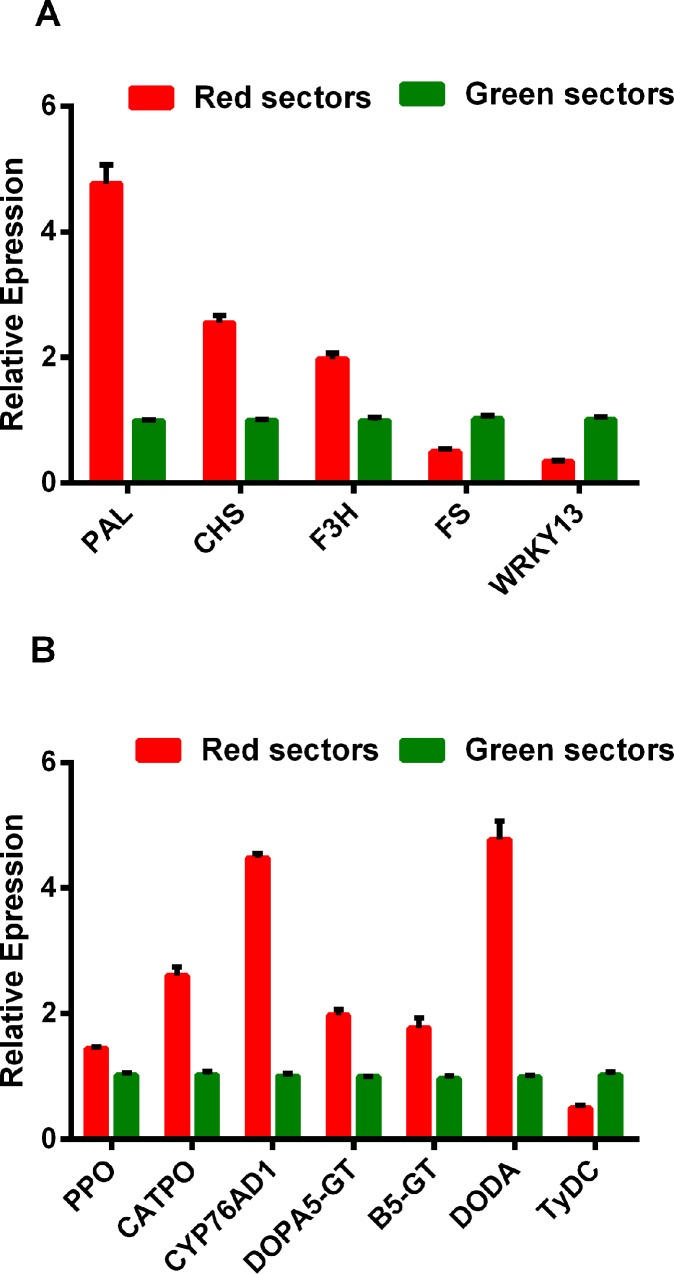
Expression levels of genes related to flavonoid and betalain metabolism in the green and red sectors of *Amaranthus tricolor* leaves. A: The expression levels of flavonoid biosynthesis genes; B: The expression levels of betalain metabolism genes.

Meanwhile, we selected 5 and 7 genes involved in the flavonoid biosynthesis and the betalain metabolic pathways to detect the expression level by using qRT-PCR assay, respectively. The expression levels of some flavonoid biosynthesis genes, including *PAL*, *CHS*, and *F3H*, were higher in the red leaf sectors than in the green leaf sectors, while the *FS* and *WRKY13* expression levels exhibited the opposite pattern (Fig 8A). To investigate the raw material supply-and-demand relationship between betalain biosynthesis and the synthesis of other metabolites in amaranth leaves, we analyzed the flavonoid metabolic pathway as an example. We completed a qRT-PCR assay to examine the expression levels of genes involved in the betalain metabolic pathways. The expression levels of genes related to betalain metabolism, such as *CATPO*, *PPO*, *CYP76AD1*, *DODA*, *DOPA5-GT*, and *B5-GT*, were higher in the red sectors than in the green sectors. The *TyDC* gene expression levels exhibited the opposite pattern. These results implied that betalain biosynthesis differs between the red and green leaf sectors of amaranth plants (Fig 8B).

## Discussion

### The conversion of tyrosine to L-DOPA during betalain biosynthesis in amaranth plants may require a complex enzyme-mediated process

Polyphenol oxidases are copper-containing redox enzymes; examples include tyrosinase (EC.1.14.18.1), catechol oxidase (EC.1.10.3.2), and laccase (EC.1.10.3.1) [60] Of these PPOs, tyrosinase, which is a special type of PPO, is believed to be active during the first two steps of betalain biosynthesis. Specifically, it catalyzes the hydroxylation of L-tyrosine to generate L-DOPA[41]. after which it catalyzes the oxidation of L-DOPA to produce dopaquinone. [61] cloned the *BvcTYR* gene, which encodes the tyrosinase responsible for the hydroxylation of L-tyrosine, and determined that this tyrosinase is involved in the first step of the betalain biosynthetic pathway.[62] recently hypothesized that low tyrosinase activity explains the relatively low betalain content of yellow beets. Two genes encoding copper-containing PPOs mediating betalain accumulation have been cloned from cultured cells [63]. Tyrosinases exhibiting *in vitro* tyrosine hydroxylase and/or DOPA oxidase activities were subsequently purified from red beet roots (Gandía-Herrero et al. 2004), Swiss chard leaves [64], and S. *salsa* seedlings[65]. However, there is still a lack of conclusive genetic evidence that tyrosinase is involved in betalain biosynthesis. In this study, we also observed that the *PPO* expression level is consistent with the betalain contents in the red and green leaf sectors. We believe that tyrosine is optimally used in the red leaf sectors because *PPO* is more highly expressed in the red leaf sectors than in the green leaf sectors. In fact, tyrosinases are widely distributed throughout the plant kingdom, including in betalain-producing Caryophyllales species. These tyrosinases still need to be comprehensively characterized in future studies.

The AcCATPO enzyme in *Amaranthus cruentus* was recently purified by Teng et al.[59]. This enzyme exhibits catalase and tyrosinase activities, and it is able to catalyze reactions involving a monophenol (tyrosine) and a diphenol (L-DOPA). Consequently, Ac*CATPO* may be important for betalain biosynthesis, especially during the first two steps of the betalain biosynthetic pathway. Furthermore, a positive correlation between *AcCATPO* transcript abundance and the ratio of betaxanthins to betacyanins implies that AcCATPO might be mainly involved in the biosynthesis of betaxanthins. However, we observed that *CATPO* is more highly expressed in the red leaf sectors than in the green leaf sectors, which is consistent with the ratio of betacyanins to betaxanthins. Because CATPO catalyzes the conversion of tyrosine to L-DOPA, which is involved in the production of both betacyanins and betaxanthins, we speculate that CATPO mediates the biosynthesis of both betacyanins and betaxanthins.

Previous studies of betalain biosynthesis in beets identified three new cytochrome P450 enzymes, namely CYP76AD1, CYP76AD5, and CYP76AD6, which hydroxylate tyrosine (monophenolase activity) [43]. These three enzymes reportedly contribute to the initiation of the betalain biosynthetic pathway in tobacco, potato, tomato, and eggplant [66]. In the current study, we identified a cytochrome P450 gene (*CYP76AD1*) in the amaranth database. The expression levels of this gene were consistent with the betalain contents in the red and green leaf sectors. Although tyrosinase is thought to be the enzyme involved in the initial step of betalain biosynthesis, future studies will need to confirm which enzyme (or enzymes) is involved in this step. There are studies that have suggested tyrosine originated from pre-tyrosine or tyramine [67, 68]. On the basis of the data presented herein, we speculate that there are multiple PPO enzymes or other enzymes involved in the conversion of tyrosine to L-DOPA in the betalain biosynthetic pathway of amaranth plants.

### A transcriptome analysis and qRT-PCR verification can increase the accuracy of the analyses of amaranth metabolic activities

Amaranth plants are an important source of vital dietary components [19–21], including betalains, flavonoids, alkaloids, and other metabolites [25–28]. Photosynthesis provides the organic materials, energy, and oxygen required for almost all life activities, including secondary metabolism. In this study, we first constructed a betalain transcriptome database, which we then analyzed to detect the various amaranth metabolic pathways, including those related to phenylpropane synthesis (35 unigenes), plant hormone signal transduction (22 unigenes), phenylalanine metabolism (22 unigenes), flavonoid biosynthesis (14 unigenes), photosynthesis (including antenna proteins) (25 unigenes), and betalain metabolism (1 unigene). Thus, our analysis of the amaranth transcriptome revealed many of the metabolic pathways in amaranth plants. Additionally, the *DODA* expression level, which influences betalain production, differed significantly between the red and green leaf sectors. However, according to Teng [59] and Zheng et al [52], We also identified unigenes associated with betalain metabolism based on comparisons of homologous gene sequences. The unigenes that were differentially expressed between the red and green leaf sectors were identified based on the following criteria for unique sequence reads: FC ≥ 2 and FDR < 0.01. The expression levels of *CATPO*, *PPO*, *MYB*, *CYP76AD1*, *DODA*, *DOPA5-GT*, *B5-GT*, and *TyDC* were significantly different between the red and green leaf sectors. The expression level of only *TyDC* was down-regulated in the red leaf sectors relative to the corresponding expression level in the green leaf sectors. The expression levels of all other genes exhibited the opposite pattern. We speculated that betalains in leaves accumulate only in the epidermal subcells of abaxial leaves, which explains the lack of difference in the expression of some genes between the red and green leaf sectors. Moreover, our results suggest there is also no difference in the flavonoid, chlorophyll, and carotenoid contents between the two examined leaf sectors. Furthermore, the expression levels of the other unigenes, as determined by qRT-PCR, were largely consistent with the data in the transcriptome database, verifying the reliability of the transcriptome database. Combining the transcriptome and qRT-PCR analyses increased the accuracy of our investigation of amaranth metabolic activities. Additionally, the MYB transcription factors involved in betalain metabolism have been cloned and subsequently verified in a qRT-PCR assay [69].

### Hormones influencing the synthesis and accumulation of betalains

There are many external factors influencing the synthesis and accumulation of betalains, especially light and hormones. Betalain biosynthesis is differentially affected by various hormones, including IAA, ABA, and 6-BA. In this study, unigenes related to plant hormones were expressed in the red leaf sectors of *A*. *tricolor* plants, although these hormones are also present in the green leaf sectors. Previous studies revealed that plant hormones increase, decrease, or have no effect on betalain turnover. For example, ABA can inhibit cell growth and betalain accumulation in *Phytolacca americana* by decreasing the availability of a betalain precursor (i.e., free tyrosine) [70]. Other studies demonstrated that 6-BA can promote betalain biosynthesis in some plant species, including *A*. *tricolor r*[52], *S*. *salsa*[71], These studies indicated that betalain metabolism may be regulated by 6-BA *via* changes to the *DODA* expression level. Several studies have verified *DODA* as a key gene for betalain metabolism. Moreover, GA can maintain skotomorphogenesis [72], implying it might indirectly inhibit betalain biosynthesis [73]. We previously reported that GA_3_ negatively regulates betalain accumulation in amaranth seedlings, and this effect is more pronounced at high GA_3_ concentrations. Furthermore, 2,4-D reportedly may induce the accumulation or degradation of betalains [74–77]. In the current study, an analysis of a transcriptome database resulted in the identification of many unigenes related to hormone metabolism as well as flavonoid and alkaloid pathways. These observations were related to pathway interactions and source–sink relationships associated with betalain metabolism.

### Many metabolic pathways co-exist in the *A*. *tricolor L.*

**Fig 9:**
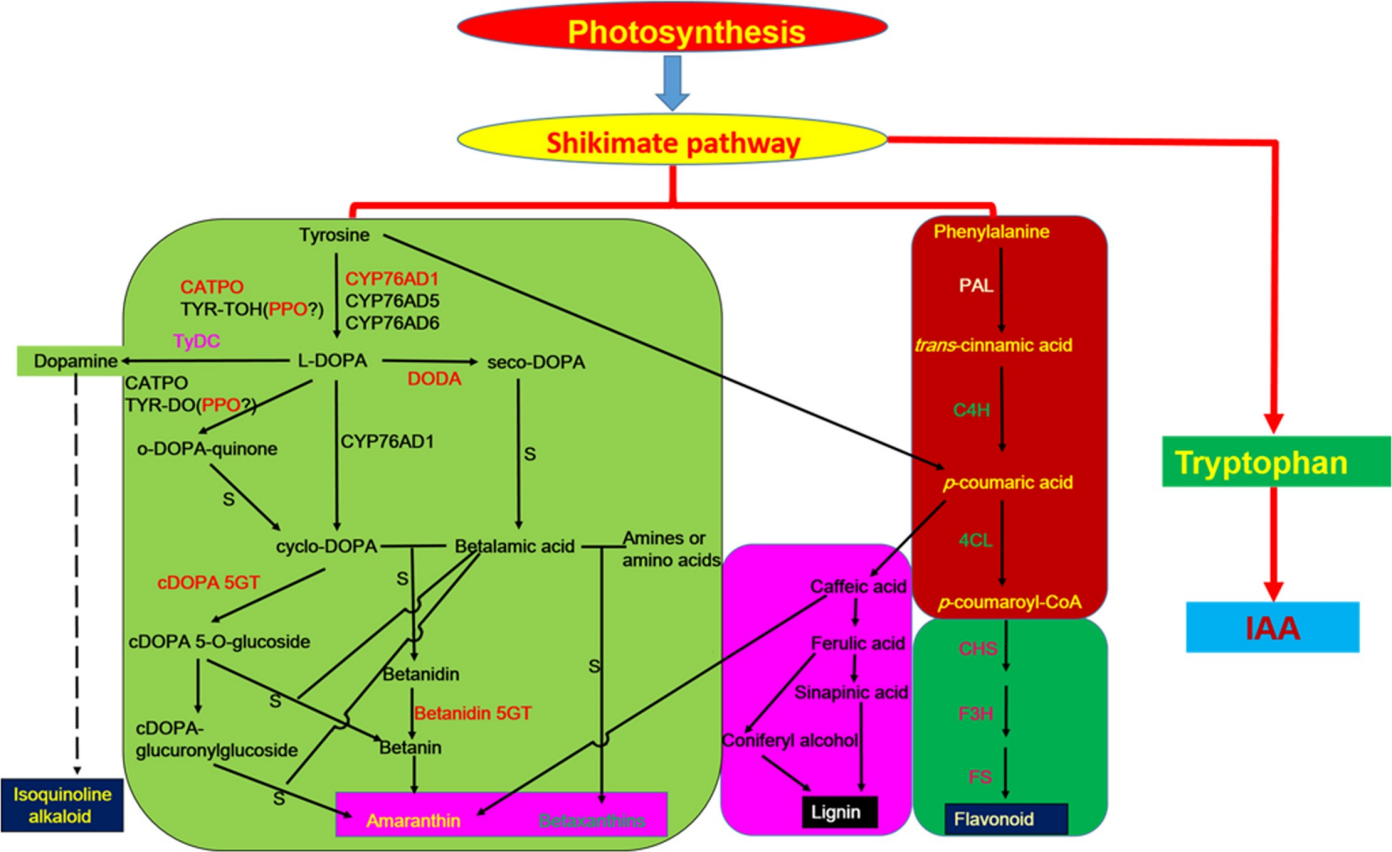
Schematic diagram of the network comprising the betalain metabolic pathway and other metabolic pathways in *Amaranthus tricolor* leaves.

Betalains have been sub-divided into betacyanins and betaxanthins. There are four main types of betacyanins (amaranthin, betanin, gomphrenin, and decarboxybetanin), of which amaranthin is the main betacyanin among *Amaranthus* species. In amaranth plants, betalain biosynthesis involves multiple metabolic pathways (Fig 9). Photosynthetic products form aromatic amino acids, including tyrosine, phenylalanine, and tryptophan, *via* the shikimic acid pathway. Tyrosine is required for protein biosynthesis, while also serving as a precursor of some metabolites, including plastoquinone, tocopherols, rosmarinic acid, isoquinoline alkaloids, catecholamines, and betalains [62]. The classic betalain metabolic pathway involves a series of enzymes, such as PPO, CYP76AD1/5/6, DODA, and glucosyltransferase. However, some reactions in this pathway occur spontaneously.

Betalains are secondary metabolites derived from L-tyrosine, which is hydroxylated to L-DOPA through the monophenolase activity of tyrosinase (or polyphenoloxidase). The L-DOPA is subsequently oxidized to dopaquinone by the diphenolase activity of tyrosinase, and then spontaneously converted to cyclo-DOPA [40]. The L-DOPA can also be directly converted to cyclo-DOPA in a reaction catalyzed by CYP76AD1-α. Additionally, L-DOPA can be converted to seco-DOPA by DODA (4,5-DOPA-extradiol dioxygenase) and then spontaneously form betalamic acid, which is a chromophore in all betalains. The addition of an amine or amino acid to betalamic acid may result in the production of yellow betaxanthin [78]. Betalamic acid may also combine with cyclo-DOPA to spontaneously form betanidin, which is a key intermediate of betacyanin biosynthesis [79]. Glucose is added to the 5-hydroxyl group of betanidin in a reaction catalyzed by betanidin-5-*O*-glucosyltransferase to form betanin, which is responsible for the characteristic color of red beets (*Beta vulgaris* ssp.). A previous study confirmed that stress can increase betalain production and up-regulate *B-5GT* expression in red beet leaves, while betalain accumulation is inhibited by the transient expression of *B-5GT* in the antisense orientation [80]. Thus, B-5GT is important for betalain metabolism. Additionally, cDOPA 5-*O*-glucosyltransferase converts cyclo-DOPA to cDOPA 5-*O*-glucoside, which combines with betalamic acid to spontaneously form betanin. The betanin is subsequently converted to amaranthin, which is the main pigment in amaranth plants. Amaranthin may also be synthesized in other pathways. For example, cDOPA 5-*O*-glucoside can be transformed to cDOPA-glucuronylglucoside and then to amaranthin. In this study, we screened the transcriptome database to identify the key genes in the betalain metabolic pathway, and we observed that the gene expression tendencies were associated with betalain content.

Amaranthin may also be synthesized *via* phenylpropane and phenylalanine metabolic activities. The metabolites might be precursors related to flavonoid and lignin metabolism. The fact that PAL and C4H can convert phenylalanine to *p*-coumaric acid suggests there is a link between betalain biosynthesis and flavonoid metabolism. The *p*-coumaric acid can be subsequently converted to ferulic acid, caffeic acid, and sinapic acid. The dihydroindoles of betacyanins might be derived from dopamine precursors, phenylalanine and tyrosine. These precursors are converted to *trans*-cinnamic acid, caffeic acid, and *p*-coumaric acid, which are important substances that link phenylpropane metabolism to flavonoid metabolism [44]. The increasing conversion of *p*-coumaric acid to amaranthin decreases the precursors available for flavonoid biosynthesis, thereby affecting the flavonoid content. Meanwhile, lignin synthesis is inhibited by the increasing use of tyrosine and phenylalanine in the pathway that transforms L-tyrosine to DOPA and then amaranthin [18]. We observed that *PAL* is more highly expressed in the red leaf sectors than in the green leaf sectors, indicating that the production of caffeic acid, which may provide the dihydroindoles in amaranthin, increases with increasing *p*-coumaric acid levels during phenylpropane metabolism. Meanwhile, we determined that *CHS* and *F3H* expression levels are higher in the red leaf sectors than in the green leaf sectors, potentially leading to a greater availability of flavonoid precursors in the red leaf sectors. These differences may be responsible for the diversity in the flavonoid production of the two leaf sectors. In contrast, the *FS* gene expression level is lower in the red leaf sectors than in the green leaf sectors, which may affect the types of flavonoid that are biosynthesized. In this study, there were no differences in the total flavonoid contents of the red and green leaf sectors. Our findings suggest that phenylalanine metabolites are not only precursors of flavonoid or lignin metabolic activities, they may also contribute to amaranthin biosynthesis.

Dopamine is a betalain metabolic intermediate that is involved in the production of betalains and isoquinoline alkaloids, which are derived from L-tyrosine. Following a series of enzymatic reactions, L-tyrosine is converted to dopamine, which is transformed into an isoquinoline alkaloid and is involved in betalain biosynthesis. Although isoquinoline alkaloids exist in amaranth plants, the relationship between isoquinoline alkaloids and betalains remains unclear.

## Conclusions

In amaranth plants, betalain metabolism is associated with a group of metabolic pathways. Additionally, multiple source–sink relationships exist between betalain biosynthesis and the synthesis of other metabolites.

## Materials and methods

### Materials

Amaranth (*A*. *tricolor* cv. Dahong) was grown on garden soil in pots in the greenhouse (28±2°C), located in the campus of Fujian Agriculture and Forestry University, China.

**Fig 10:**
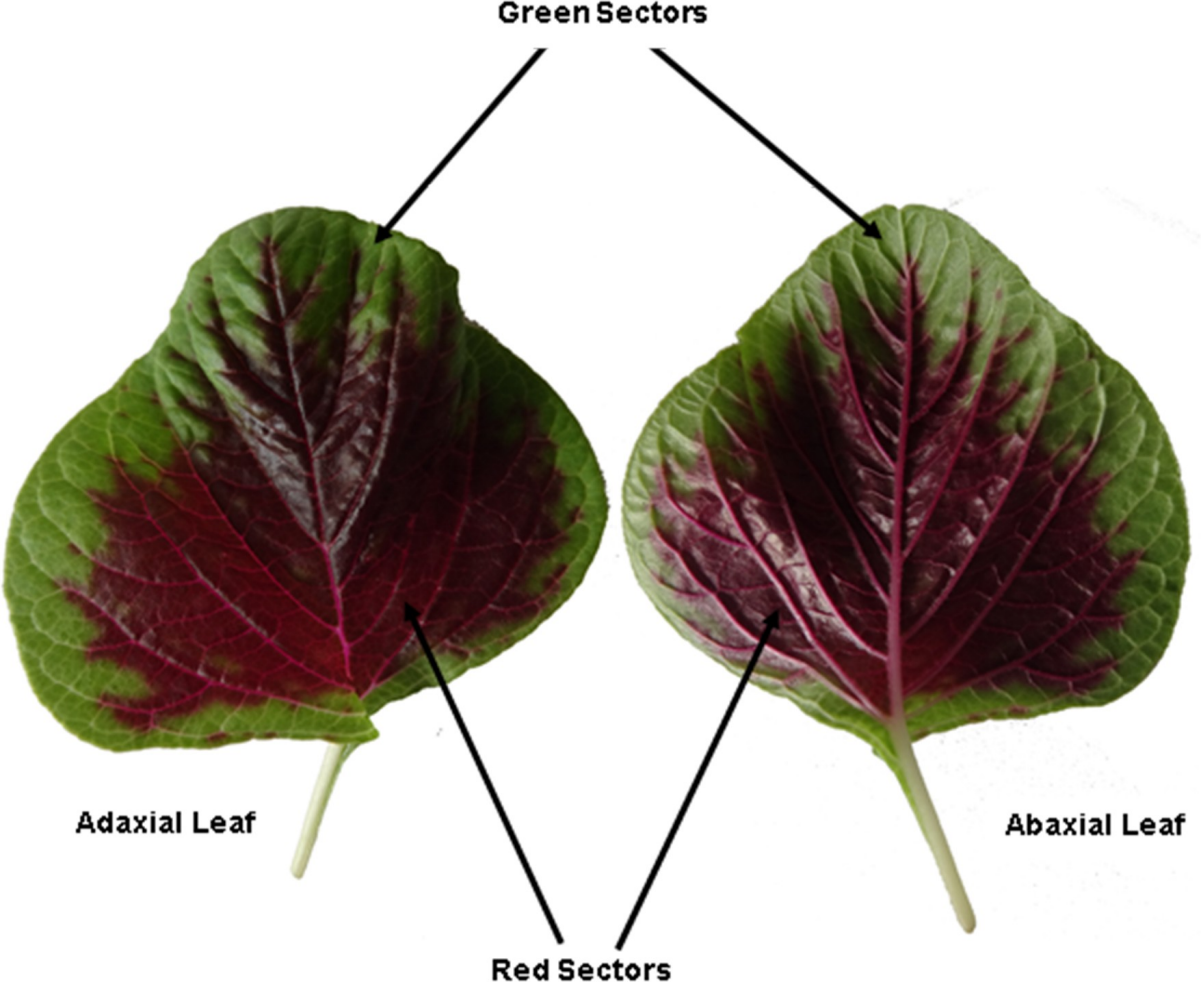
The leaf of *A*. *tricolor*.

Leaves at the fourth and fifth positions were collected from ten 30-day-old amaranth seedlings (Fig 10). The green and red sectors without veins from each seedling were respectively collected for RNA extraction and determination of chlorophyll, carotenoid, betalain, and flavonoid contents.

### Determination of chlorophyll, carotenoid, betalain, and flavonoid contents in amaranth leaves

The chlorophyll, carotenoid, betalain, and flavonoid contents in the red and green leaf sectors of amaranth plants were determined. Chlorophyll and carotenoid contents were analyzed as described by [81], Meanwhile, the betalain and flavonoid contents were determined as described by [52] and Li et al. [82], respectively.

### Total RNA extraction and quality detection

The RNA of all the red sectors or green sectors from the ten seedlings was mixed respectively. Total RNA was extracted from the collected leaf samples as previously described [47]. Specifically, the RNA was extracted with Trizol reagent (Invitrogen, USA) and treated with DNase I to eliminate any residual genomic DNA. The extracted RNA was quantified with the NanoDrop 2000 spectrophotometer (Thermo, USA), and its integrity was assessed with the Agilent 2100 Bioanalyzer (Agilent Technologies) and by denaturing agarose gel electrophoresis with ethidium bromide staining. Each RNA sample concentration and the amount was ≥ 300 ng/μL and 15 μg, respectively.

### Construction, detection, and sequencing of transcriptome libraries

A cDNA library was constructed for each sample according to the ‘Preparing Samples for Sequencing of mRNA Test Kits’ instructions (Illumina). The cDNA library quality and fragment lengths were evaluated with the Agilent 2100 DNA 1000 Kit, after which the cDNA libraries were sequenced with the Illumina HiSeq 2500 system (125-bp paired-end reads).

### Sequencing data assembly and gene annotation analysis

The raw data generated from the sequencing of each cDNA library were transformed into sequence data (i.e., raw data or raw reads) by base calling. Adapter fragments, duplicated sequences, low-quality reads with ambiguous bases (“N”), and reads with more than 10% of bases with a Q-value < 30 were removed from the raw reads to yield the clean reads for subsequent analyses. The clean reads were included in a *de novo* transcriptome assembly, which was completed with the Trinity program. Firstly, the sequencing reads were broken into shorter fragments (K-mer) using Trinity software. These shorter fragments were combined to form longer fragments (i.e., contigs) with no ambiguous basesby overlapping reads of a k—1 nucleotideslength Thirdly, by using the overlap between these longer fragments, the component was obtained. Finally, the De Bruijn graph method and sequencing read information were used to identify transcripts in each component. The resulting sequences were designated as unigenes. The ORFs of unigene sequences longer than 200 bp were identified with the getorf program (http://emboss.bioinformatics.nl/cgi-bin/emboss/getorf). The unigene sequences were then used as queries in a BLASTX search (*E*-value < 1 × 10^−5^) of the NCBI non-redundant (Nr), Swiss-Prot, GO, Clusters of Orthologous Groups (COG), euKaryotic Orthologous Groups (KOG), and KEGG databases. The enriched KEGG pathways among the unigenes were identified with the KOBAS 2.0 program. We used the HMMER program to compare the predicted amino acid sequences encoded by unigenes with sequences in the Pfam database to functionally annotate the unigenes.

### Analysis of differentially expressed genes

The Bowtie program was used to compare the reads for each sample with the sequences in a unigene library. On the basis of this comparison, gene expression levels were estimated with the RSEM v1.3.0 program (2016). The FPKM (fragments per kilobase of transcript per million mapped reads) value was used to represent unigene expression levels. After normalizing the unigene expression levels, genes that were differentially expressed between the red and green leaf sectors were identified with the DESeq program. A *p*-value < 0.05 was applied as the threshold after the adjustment for multiple comparisons based on the Benjamini and Hochberg false discovery rate (FDR) method. The unique reads with a fold-change (FC) value ≥ 2 and FDR < 0.01 were identified as DEGs.

We used the MapMan 3.5.1R2 program to compare the unigene sequences with *Arabidopsis thaliana* metabolism pathways. Some of the matching genes related to flavonoid metabolism were selected for further study. Additionally, the betalain-related genes (i.e., associated with the betalain biosynthetic pathway) were also selected.

### Quantitative real-time polymerase chain reaction analysis

Ten DEGs were analyzed in a qRT-PCR assay to validate the accuracy of the RNA-sequencing (RNA-Seq) data. The genes related to betalain biosynthesis and flavonoids were then analyzed. Gene-specific primers designed with the DNAMAN 6.0 program (LynnonBiosoft, America) were synthesized by Shanghai Bio-engineering Co., Ltd. Details regarding the primers are presented in S5A and S5BA Table.

The RNA samples used to construct cDNA libraries were used for the qRT-PCR analysis, which was completed with the SYBR Green I Master Mix (Takara) and the LightCycler 480 qRT-PCR instrument (Roche, Switzerland). All samples were analyzed in triplicate, with three biological replicates per sample. The *EF1a* gene was used as an internal reference for calculating relative unigene expression levels. Specific details regarding the qRT-PCR method were previously described[47].

## Statistical analysis

Quantitative results of the gene expression and component contents analyses were presented in terms of means ± SDs of at least three biological replicates. The gene expression and component contents were analysed by one-way analysis of variance (ANOVA) followed by Duncan’s test using SPSS version 19.0. These pictures were made using the GraphPad Prism 6.0 software and Excel 2013.

## Supporting information

S1 Table Top 10 significantly altered GO enrichment related pathways identified in the process of betalains metabolism. (XLS)

S2 Table Hormone metabolism related DEGs between red and green sectors of *A*. *tricolor* leaf. (XLS)

S3 Table Photosynthesis DEGs between red and green sectors of *A*. *tricolor* leaf. (XLS)

S4 Table Sucrose and starch metabolism related DEGs between red and green sectors of *A*. *tricolor* leaf. (XLS)

S5 Table Table a Primers for validating the accuracy of RNA-Seq data and betalains related metabolisms.
Table b Primers used for gene expression analysis related to betalains and flavonoid by qRT PCR.
(XLS)

S1 Fig Amaranthus Unigene length distribution. (TIF)

S2 Fig COG categorie. (TIF)

## Abbreviations

B5-GT: betanidin 5-*O*-gluc
osyltransferaseTyDC: tryptophan decarboxylase
C4H: cinnamate 4-hydroxylase
CATPO: catalase-phenol oxidase
CHS: chalcone synthase
CYP76AD1: cytochrome P450, CYP76AD1 is a member of cytochrome P450
DODA: DOPA 4,5-dioxgenase
DOPA: 3,4-dihydroxyphenylalanine
DOPA5-GT: cyclo-DOPA 5-O glucosyltransferase
F3H: flavanone 3-O hydroxylase
FS: flavonol synthase
GO: Gene Ontology
KEGG: Kyoto Encyclopedia of Genes and Genomes
NAD(P): Nicotinamide adenine dinucleotide (phosphate)
ORF: open reading frame
PAL: phenylalanine ammonia-lyase
PPO: polyphenol: oxidase
qRT-PCR: real-time quantitative PCR
TYR: tyrosinase
WRKY13: WRKY-type transcription factor 13
