# Supplementary figures and images for: RNA-sequencing analysis reveals betalains metabolism in the leaf of Amaranthus tricolor L
Source: PLoS One. 2019 Apr 25;14(4):e0216001. doi: 10.1371/journal.pone.0216001 (PMC6483260; doi:10.1371/journal.pone.0216001)

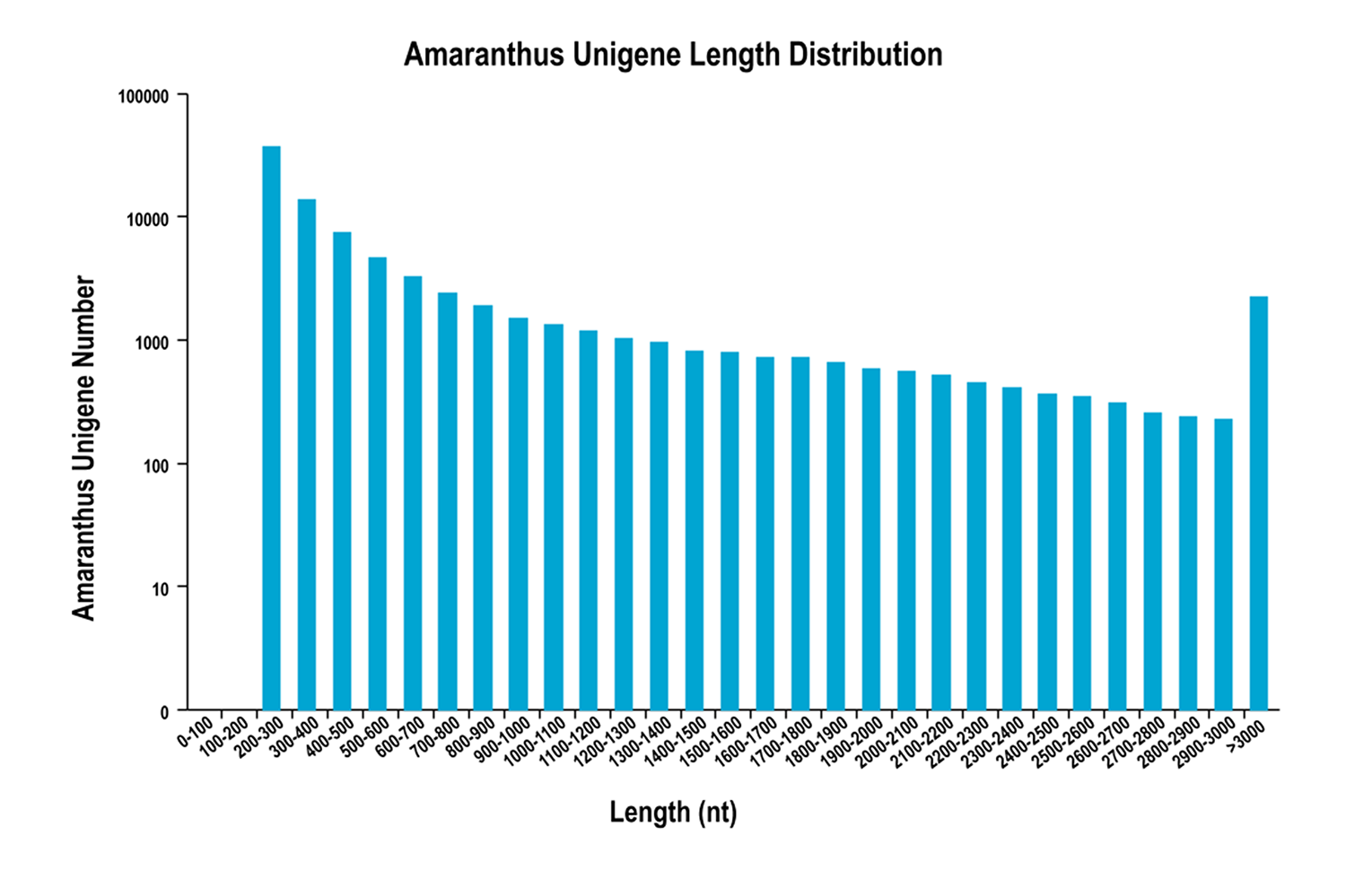

Supplement: S1 Fig — (TIF) [file pone.0216001.s006.tif]

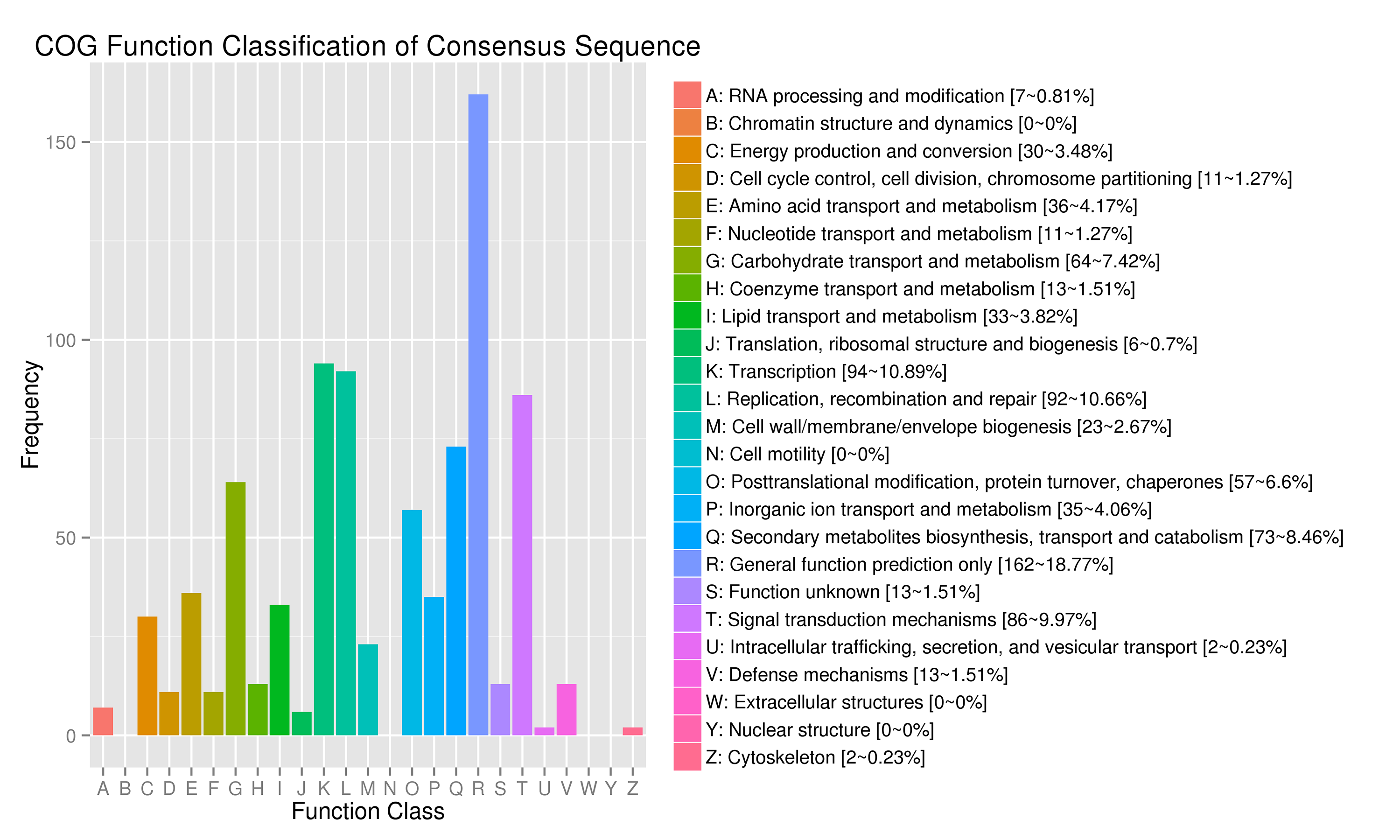

Supplement: S2 Fig — (TIF) [file pone.0216001.s007.tif]
